# Practitioners of Medicine in Boston

**Published:** 1851-11

**Authors:** 


*Practitioners of Medicine in Boston.* — Boston, with a population of 130,000, numbers 350 practitioners of medicine, of all shades and stripes. Of this number, 213 belong to the State Medical Society; 51 are unconnected with any medical association; 20 are botanic physicians, and 6 *female physicians!* Nothing is said as to the number of homœopathic practitioners, but we presume they are included among the 51 *outsiders,* as it is hardly to be supposed that Boston is free from that species of professional nuisance.— *St. Louis Med. Journal.*

